# Surgeon-Administered Nerve Block During Rotator Cuff Repair Can Promote Recovery with Little or No Post-operative Opioid Use

**DOI:** 10.1007/s11420-019-09745-4

**Published:** 2020-01-22

**Authors:** George L. Caldwell, Michael A. Selepec

**Affiliations:** Caldwell Sports Medicine, 2307 West Broward Blvd., Suite 200, Fort Lauderdale, FL 33312 USA

**Keywords:** rotator cuff repair, shoulder arthroscopy, opioids, suprascapular nerve, axillary nerve, shoulder nerve block, sports medicine

## Abstract

**Background:**

The use of opioid analgesia is common in both the acute and extended post-operative periods after rotator cuff repair. The current opioid crisis has prompted surgeons to seek alternatives that minimize or even eliminate the need for oral opioids after surgery.

**Questions/Purposes:**

We sought to investigate the effects on post-operative opioid use of a surgeon-administered block of the suprascapular and axillary nerves in arthroscopic rotator cuff repair (ARCR), in particular to quantify outpatient opioid consumption and duration.

**Methods:**

In this prospective observational study, all patients undergoing primary ARCR performed under general anesthesia by a single surgeon were studied over a 15-month period. Of 91 ARCRs performed, 87 patients were enrolled and followed prospectively. At the conclusion of the procedure, the surgeon performed “local–regional” nerve blockade with injections to the sensory branches of the suprascapular nerve and the axillary nerve, as well as local infiltration about the shoulder. Use of medications in the post-anesthesia care unit was left up to the anesthesiologist. Patients were prescribed oral opioids (hydrocodone/acetaminophen 5/325 mg) for analgesia after discharge. The quantity and duration of opioid use and pain scores were recorded for 4 months. Statistical analysis was performed to evaluate factors that could account for greater opioid use.

**Results:**

Total opioid consumption ranged from 0 to 30 opioid tablets (average, 4.2 tablets) over the 4-month period. Post-operatively, 91% of patients took between ten or fewer tablets, and 39% took no opioids. The average duration of opioid use was 2.4 days. No patients were taking opioids at the 4- to 6-week or 4-month follow-up visits, none required refills, and none received prescriptions from outside prescribers. No statistically significant differences were seen in opioids taken or duration of use in regard to tear size, sex, body mass index, surgery location, or procedure time. There was a significant inverse correlation between opioid use and age. In addition, the cost of the surgeon-performed procedure was substantially lower than that associated with pre-operative nerve blockade performed by an anesthesiologist. All patients were satisfied with the post-operative pain management protocol. Average reported post-operative pain scores were low and decreased at each visit.

**Conclusion:**

With this local–regional nerve-blocking protocol, opioid use after ARCR was unexpectedly low, and a large proportion of patients recovered without any post-surgical opioids. The correlation seen between opioid use and age may not be clinically significant, given the low use of post-operative opioids overall. These results may be useful in guiding post-operative opioid prescribing after ARCR, as well as in lowering costs associated with ARCR.

**Electronic supplementary material:**

The online version of this article (10.1007/s11420-019-09745-4) contains supplementary material, which is available to authorized users.

## Introduction

Outpatient arthroscopic rotator cuff repair (ARCR) is a common procedure, but it has been associated with significant post-operative pain [15, 24]. Effective post-operative pain management is especially crucial in terms of recovery time, rehabilitation goals, and patient satisfaction. However, both new and persistent opioid use pose previously underappreciated risks of surgical complications [3]. Recently, long-term opioid use among opioid-naïve patients was reported in 13.9% of patients after ARCR [12]. A database study of surgical patients without a history of misuse or ongoing opioid use revealed a 44% increase in misuse for every opioid refill [2].

There has been a call for evidence-based guidelines for prescribing opioids after orthopedic surgery [2]. Studies of anesthesia that focus on the first days after surgery may not provide insight into patients’ medication use during the months of rehabilitation and recovery after ARCR. A recent review determined that a major obstacle to developing such guidelines was the “lack of data on post-discharge use of opioids as well as the paucity of studies focusing directly on recording patterns in post-operative opioid consumption” [13].

Surgeons are searching for ways to improve opioid stewardship without compromising effective analgesia for ARCR. Regional nerve blocks are widely used in ARCR to control acute surgical pain [8]. Interscalene nerve block (ISB), which has consistently been shown to reduce early post-operative pain [1], has been considered the gold standard. However, there are a number of possible complications with ISB, including nerve damage, intravascular injection, diaphragm dysfunction with respiratory compromise, and pneumothorax [1]. Other blocks that, like ISB, are used in conjunction with neurostimulation or ultrasound guidance include suprascapular nerve block (SSNB) and axillary nerve block (ANB) [18]. The safety and efficacy of surgeon-administered combined SSNB and ANB for shoulder arthroscopy have been demonstrated [5, 17].

In 2019, we published a study in this journal on the effects of surgeon-administered local and regional blockade (“local–regional block”) of specific genicular nerves on post-operative analgesia after anterior cruciate ligament reconstruction (ACLR); we quantified outpatient opioid consumption and duration through the complete post-operative course [4]. We found post-operative opioid use to be unexpectedly low in our patient population, and the overwhelming majority of patients were “strongly satisfied” with their surgery.

The purposes of the current study were similar: to investigate the efficacy of a surgeon-administered local–regional block of specific nerves on post-operative analgesia after ARCR in opioid-naïve patients and to quantify the outpatient opioid consumption and duration throughout the post-operative course. We hypothesized that a new protocol of surgeon-administered local–regional anesthetic to the suprascapular and axillary nerves could provide desirable blockade with sufficient post-operative pain control while lowering post-operative oral opioid requirements. We also aimed to answer questions about long-term (at 4 months) opioid requirements after ARCR and differences in opioid requirements that may be associated with surgical and patient variables.

## Methods

The methods used in this study were similar to those reported in our study of post-operative opioid use after ACLR [4]. Institutional review board (IRB) approval was obtained for this prospective observational study; a waiver of consent was granted. Eligible patients were those undergoing primary ARCR performed by a single surgeon from August 2017 through October 2018. Ninety-one patients older than 18 years in whom ARCR was clinically indicated were considered for inclusion. Exclusion criteria were a history of pre-operative opioid use; revision surgery; and allergy to oral opioids, nonsteroidal anti-inflammatory drugs (NSAIDs), or local anesthetic. We defined pre-operative opioid use as having filled opioid prescriptions within 3 months before ARCR or having a history of opioid dependency. Three patients were excluded because of pre-operative opioid use, and one was excluded because of NSAID allergy. A total of 87 patients were included and followed prospectively. No patients were lost to follow-up. Data collected included pre-operative demographics and clinical characteristics, such as age, sex, and body mass index (BMI).

All procedures were performed on an outpatient basis using standardized operative and post-operative treatment protocols. Procedures were performed at two facilities (one hospital and one ambulatory surgery center), each with its own anesthesia group. According to practice protocol at both facilities, prescription of intra-operative medications was left to the discretion of the individual anesthesiologists. The patients were placed in the beach chair position after induction of general anesthesia. No pre-operative blocks were performed. All surgical procedures were completed using a standardized protocol by the study’s surgeon. An arthroscopic suture-bridge technique was used to repair the rotator cuff. This double-row technique involves the creation of a medial and a lateral row of bioabsorbable anchors (4.75 mm in diameter). The cuff was mobilized for a direct repair to the tuberosity; no margin convergence was used. The remaining parts of the procedure (acromioplasty, Mumford procedure, and biceps tenodesis or tenolysis) were then performed as necessitated by the associated pathology. The intra-operative medications provided were also at the discretion of the anesthesiologist and were not recorded.

The nerve blocks were administered at the conclusion of the procedure, in the operative suite with the patient still under general anesthesia. The injections were performed by the surgeon with the aim of providing anesthetic blocks to the sensory branches of the suprascapular nerve and axillary nerve, as well as local infiltration about the shoulder. We termed this a “local–regional block” of the shoulder. We used no ultrasound or neurostimulation to guide administration of the local–regional block. The local anesthetic infiltration included a total of 60 cc of 0.25% bupivacaine (not to exceed 2 mg/kg) using a 22-gauge needle.

**Fig. 1:**
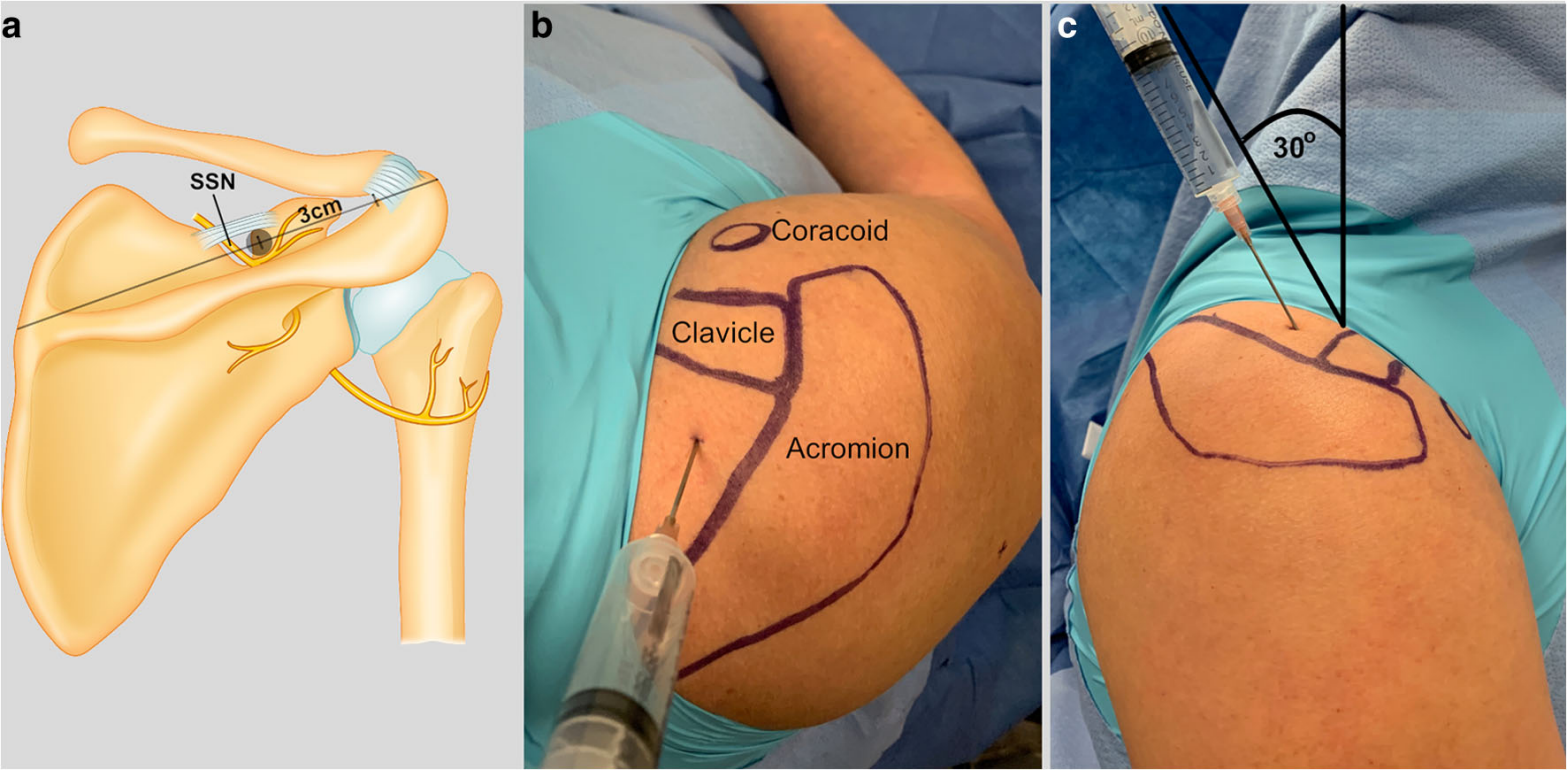
Suprascapular nerve blockade. The approach to the suprascapular nerve, **a**, was based on superficial anatomic landmarks. **b** The acromion, spine of the scapula, clavicle, and coracoid process were outlined; a line was drawn from the anterolateral edge of the acromion to the medial edge of the scapular spine; the insertion point for the needle was along this line. **c** The needle was then inclined 30° anteriorly from perpendicular in the sagittal plane of the body toward the coracoid.

The approach to the suprascapular nerve was based on superficial anatomic landmarks and similar to that described by Matsumoto et al. [14]. The skin was marked before the procedure: the acromion, spine of the scapula, clavicle, and coracoid process were outlined; a line was drawn from the anterolateral edge of the acromion to the medial edge of the scapular spine (Fig. 1). The insertion point for the needle was along this line, 3 cm medial from the medial edge of the acromion. The surgeon did make adjustments if necessary because of distortion from fluid distention. The needle was then inclined 30° anteriorly from perpendicular in the sagittal plane of the body toward the coracoid. The needle was advanced to touch the base of the coracoid, and 10 cc of anesthetic was then injected slowly. Repeated withdrawal was used to prevent intravascular injection.

**Fig. 2:**
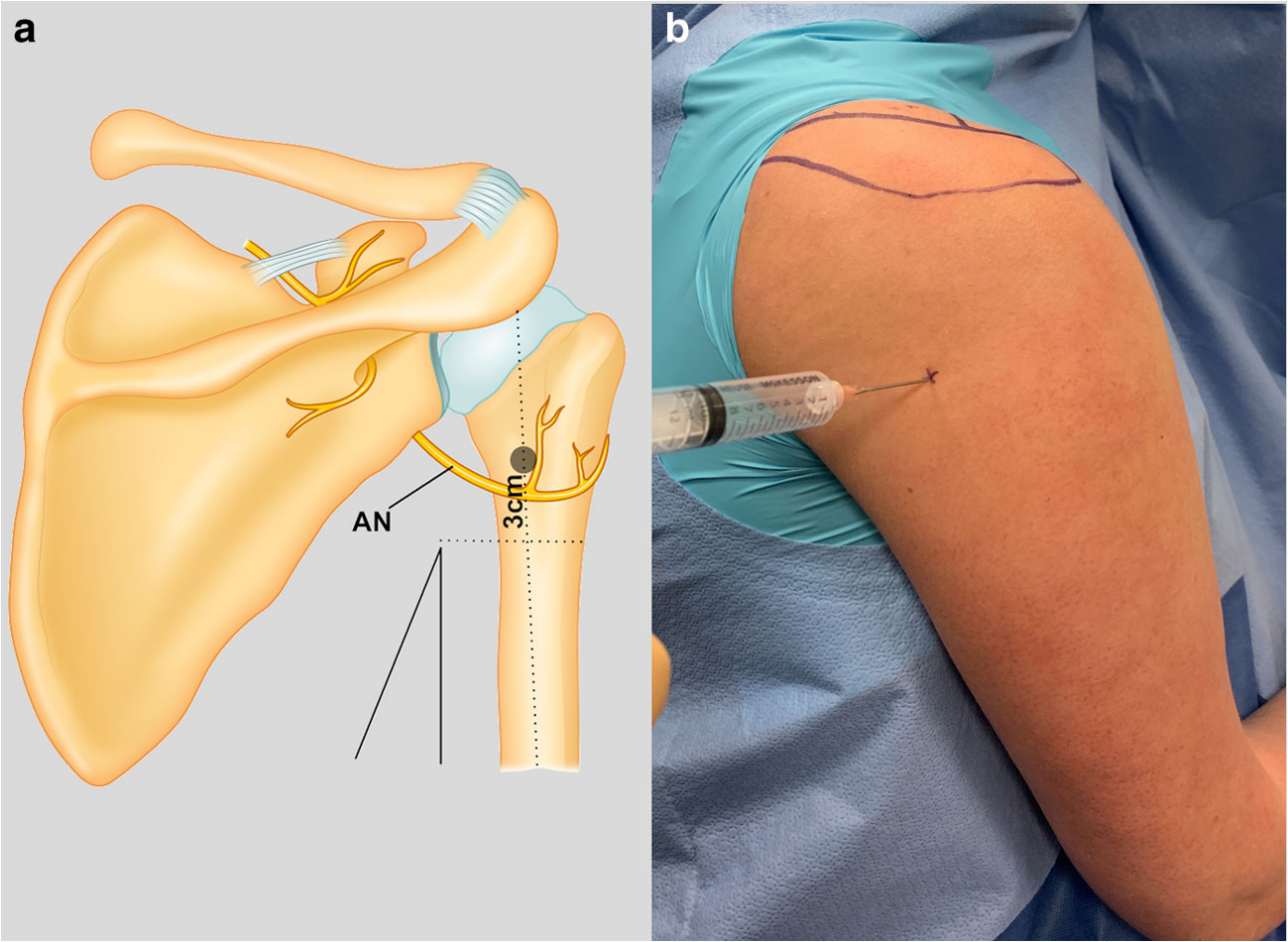
Axillary nerve blockade. A horizontal line was drawn from the posterior axillary fold to the lateral aspect of the upper arm. **a** A vertical line was then drawn from (approximately) the posterolateral corner of the acromion to the olecranon tip. **b** The needle was then inserted 3 cm cranial to the convergence of these lines until it came in contact with the posterior cortex of the proximal humerus.

ANB was then performed. Our approach was similar to the method described by Park et al. [17]. Posteriorly, a horizontal line was drawn from the posterior axillary fold to the lateral aspect of the upper arm. A vertical line was then drawn approximately from the posterolateral corner of the acromion to the olecranon tip (Fig. 2a). The needle was then inserted 3 cm cranial to the convergence of these lines until it came in contact with the posterior cortex of the proximal humerus (Fig. 2b). Then, 10 cc of anesthetic was slowly injected after repeated withdrawals. The remaining anesthetic was injected carefully into the subadipose layer above the fascia over the anterior acromion and acromioclavicular joint and medial to the anterior and posterior incisions. No intra-articular glenohumeral injection was performed.

After wound closure, the dressing and sling with abduction pillow were applied in the operating room, and the patient was transferred to the post-anesthesia care unit (PACU). Patients were given ice packs and encouraged to use them frequently. Additional pain medication was administered through the intramuscular, intravenous, or oral route according to the patient’s pain level and the anesthesiologist’s discretion. Although the medications and dosages used varied between anesthesia groups, adjunctive medication was limited to short-acting opioids, acetaminophen, and NSAIDs. No long-acting medication, ketamine, or gabapentin was administered. Additional pain pumps, nerve blocks, and patient-controlled analgesia (PCA) were not used. Surgical time, PACU hold time, and facility were recorded for future comparison.

In accordance with our usual practice, patients were discharged with a prescription for 30 tablets of hydrocodone/acetaminophen 5/325 mg (5 oral morphine equivalents [OMEs] per tablet) [6]. All patients were also given a prescription for 20 tablets of ibuprofen 600 mg. The recommendation was to take an ibuprofen tablet twice daily with meals for pain. If the pain was not adequately controlled (subjectively), patients could take a hydrocodone/acetaminophen 5/325 mg tablet every 4 h as needed. No other medications were prescribed. Over-the-counter ibuprofen use as needed after completion of the initial ibuprofen prescription was recommended. Pre-operatively, we had discussed expectations for pain and analgesic use. It was recommended to patients that they take opioid medication only for severe breakthrough pain that was refractory to ibuprofen and ice. The definition of “severe” was not tied to a particular pain intensity score. At each post-operative visit, patients were informally encouraged to discontinue opioid pain medication as soon as, in their opinion, pain was tolerable. Patients were directed to follow the US Food and Drug Administration’s medication-disposal policy on disposing of unused opioids, in order to minimize the chances of diversion [25].

**Fig. 3:**
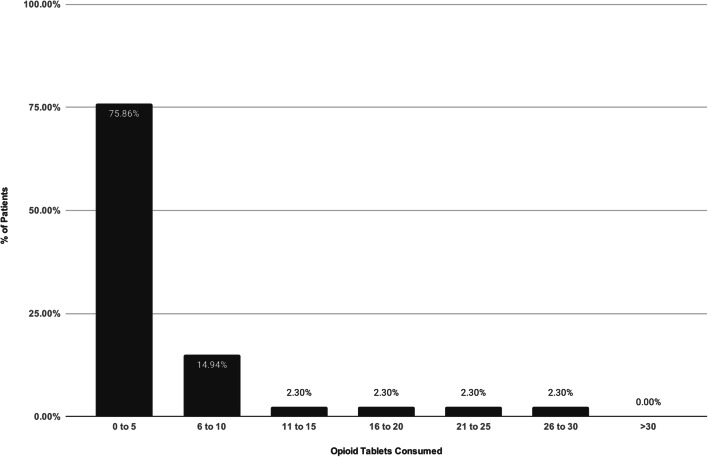
Post-operative opioid consumption—number of tablets taken. Patients were prescribed 30 tablets of 5/325 mg hydrocodone/acetaminophen.

Post-operatively, all patients were seen by the surgeon or physician assistant (PA) in the office the day after surgery, on post-operative day 7, at 4 to 6 weeks, and at 4 months. Patients were seen more frequently if it was deemed clinically necessary. Patients had a dressing change on day 1 and were allowed to shower that evening. Post-operative therapy, using a standardized protocol, began within 7 days after surgery, focusing on a return of full range of motion (ROM) and restoration of strength. Use of the sling was discontinued at 6 weeks. Physical exams at 4 to 6 weeks and at 4 months included documentation of any sensory deficit over the shoulder or perceived suprascapular or axillary nerve dysfunction on manual muscle testing.

The primary study outcome measure was post-discharge use of opioid pain medication. The quantity of opioid use (number of a hydrocodone/acetaminophen 5/325 mg tablets [± standard deviation]) and duration of opioid use (latest post-operative day on which they were taken) were documented at each visit. In addition, any prescription refill requests and visual analog scale (VAS) pain scores were recorded. Patients were specifically asked during the first two visits about rebound pain crisis. This was defined as a significant pain event causing personal distress, phone call to a physician, or evaluation at an emergency department. Patients’ satisfaction with our post-operative pain management protocol was assessed using a Likert scale at the third post-operative visit. Satisfaction was rated 1 to 5, with 1 being strongly dissatisfied and 5 being strongly satisfied.

Patients reported their opioid use (verbally) at each visit, having completed self-administered at-home pill counts. Data were entered into the patient’s electronic medical record and a spreadsheet. All patients were prescribed the same post-operative dosage of opioids. The dosage was also converted to OMEs to facilitate later comparisons with other studies. At the 6-week and 4-month visits, we accessed the Florida Prescription Drug Monitoring Program (also known as E-FORCSE®) database to determine whether any opioids had been prescribed by other physicians.

Each patient’s rotator cuff tear was measured intra-operatively and assigned to one of three categories according to the size of the tear: small (0 to 1 cm), medium (1 to 2 cm), or large (> 2 cm). Statistical analysis and graph creation were accomplished using Excel software (Microsoft Corp., Redmond, WA, USA). We performed statistical analysis of demographic and clinical factors that could contribute to greater opioid consumption or increased duration of use. Because of the nonparametric nature of our data, we used the Mann–Whitney *U* test for categorical variables, and Spearman *ρ* correlations for continuous variables. For categorical variables with more than two groups, we initially ran a Kruskal–Wallis analysis of variance. For variables with between-group values with a *p* value lower than 0.1, subgroups were evaluated pairwise using the Mann–Whitney *U* test. For all variables, *p* < 0.05 was considered to be statistically significant.

## Results

**Fig. 4:**
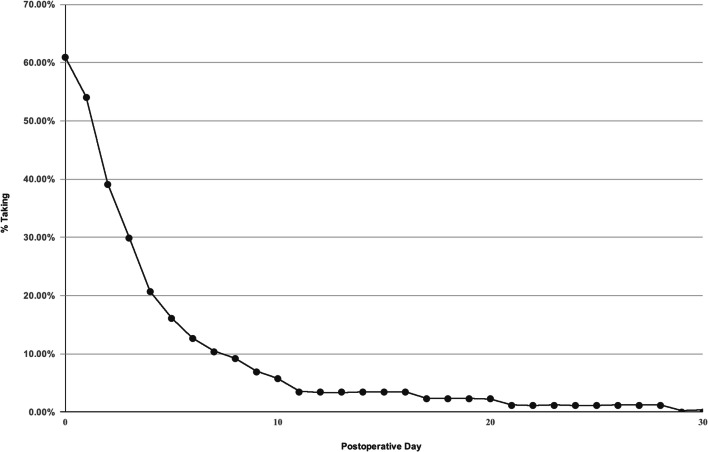
Duration of post-operative opioid use. In total, 90% of patients had discontinued opioid use by post-operative day 7. No patients took opioid medication for longer than 1 month.

Complete data were available for all patients. Table 1 shows patient demographics and clinical characteristics, the latter before, during, and after surgery. Over the 4-month post-operative course we followed, the average number of opioid tablets used per patient was 4.2 ± 6.45 (range, 0 to 30; median, 2). Overall, 91% of patients took between zero and ten tablets in total; 39% of patients took no opioid pain medication (Fig. 3). Only one patient (1.15%) took all 30 tablets. The average duration of opioid use was 2.4 ± 4.43 days (median, 1 day). In total, 90% of patients had discontinued opioid therapy by post-operative day 7 (Fig. 4). No patients took opioid medication for longer than 1 month. There were no prescription renewals. According to the E-FORCSE database, no patients received opioid prescriptions from outside physicians during the study period.

**Table 1:** Patient demographics and clinical characteristics before, during, and after surgery

Patients, no.	87
Age, years (± SD)	60.3 (± 10.2)
BMI (± SD)	28.2 (± 5.1)
Sex, male, no. (%)	56 (64%)
Site of outpatient surgery, no.	
Hospital	41
ASC	46
Procedure time, min (± SD)	95.4 (± 24.6)
Tear size, no. (%)	
Small (0 to 1 cm)	13 (14.9%)
Medium (1 to 2 cm)	42 (48.3%)
Large (> 2 cm)	32 (36.8%)
PACU time, min (± SD)	139.2 (± 78.1)
Intra-operative complications, no.	0
Post-operative complications, no.	0
VAS score, 0–10 (± SD)	
Pre-operative	4.4 (± 2.5)
PACU	2.2 (± 2.1)
Day 1	2.8 (± 2.0)
Day 7	1.3 (± 1.6)
Weeks 4 to 6	0.7 (± 1.3)
4 months	0.2 (± 0.5)
Average opioid use, no. of tablets taken (± SD)^a^	4.2 (± 6.4)
Average duration of opioid use, days (± SD)	2.4 (± 4.4)
Patient satisfaction—on a scale of 0 to 5, from strongly dissatisfied to strongly satisfied (± SD)	4.9 (± 0.1)

*SD* standard deviation, *BMI* body mass index, *ASC* ambulatory surgery center, *PACU* post-anesthesia care unit, *VAS* visual analog scale

^a^All tablets contained 5 mg hydrocodone and 325 mg acetaminophen

Patients were divided into four age groups for comparison (Table 2). There was a significant correlation between age and opioid consumption (number of tablets taken), with opioid use decreasing with age (*p* = 0.043; Table 3). However, no significant correlation between age and duration of opioid use was seen (*p* = 0.071). No significant differences were seen in opioid consumption or duration of use with the remaining variables (Table 3).

**Table 2:** Opioid use by age group

Age group	Average (± SD)
Under 50 years	
Opioid tablets taken, no.^a^	6.87 (± 6.67)
Duration of use, days	2.73 (± 2.57)
50 to 59 years	
Opioid tablets taken, no.	4.33 (± 6.70)
Duration of use, days	2.50 (± 3.77)
60 to 69 years	
Opioid tablets taken, no.	3.55 (± 6.69)
Duration of use, days	2.66 (± 6.24)
Over 70 years	
Opioid tablets taken, no.	2.89 (± 4.75)
Duration of use, days	1.79 (± 2.59)

^a^All tablets contained hydrocodone 5 mg and acetaminophen 325 mg

**Table 3:** Analysis of opioid use in relation with demographic and clinical variables

Variable	Subgroup mean (± SD)	*p* value
Age		
Opioid tablets taken, no.^a^	—	0.043
Duration of use	—	0.071
BMI		
Opioid tablets taken, no.	—	0.217
Duration of use, days	—	0.321
Sex		
Opioid tablets taken, no.		0.363
Male	4.05 (± 6.80)	
Female	4.45 (± 5.77)	
Duration of use, days		0.435
Male	2.38 (± 4.63)	
Female	2.53 (± 4.04)	
Facility		
Opioid tablets taken, no.		0.327
Hospital	3.20 (± 5.97)	
ASC	5.09 (± 6.73)	
Duration of use, days		0.582
Hospital	2.20 (± 3.90)	
ASC	2.65 (± 4.85)	
Procedure time		
Opioid tablets taken, no.	—	0.577
Duration of use, days	—	0.725
Tear size		
Opioid tablets taken, no.		0.645
Small (0–1 cm)	3.92 (± 4.60)	
Medium (1–2 cm)	3.74 (± 6.09)	
Large (> 2 cm)	4.90 (± 7.43)	
Duration of use, days		0.968
Small (0–1 cm)	2.07 (± 2.46)	
Medium (1–2 cm)	2.85 (± 5.66)	
Large (> 2 cm)	2.03 (± 2.91)	
PACU time		
Opioid tablets taken, no.	—	0.212
Duration of use, days	—	0.499

*SD* standard deviation, *BMI* body mass index, *ASC* ambulatory surgery center, *PACU* post-anesthesia care unit

^a^All tablets contained 5 mg hydrocodone and 325 mg acetaminophen

Average patient-reported pain scores on the 10-point VAS were low (≤ 3) for all post-operative visits (Table 1). There was a small increase in reported pain on post-operative day 1, as compared with pain at the time of discharge (day 0). However, at all subsequent visits, the average reported pain score was lower. All patients reported satisfaction with our post-operative pain management protocol.

During the complete post-operative interval of the study, there were no readmissions, emergency department or urgent care visits, or after-hours phone calls for opioid management. There were no complications, infections, or reoperations. There were no complications regarding the suprascapular or axillary nerve blocks (no cardiac issues related to intravascular injection, prolonged numbness, paresthesias, or motor deficits). The cost of the local–regional technique was approximately $12 (for 60 cc of bupivacaine) and generally took an additional 2 min for the surgeon to administer after surgery.

## Discussion

The principal findings of this study are comparable with those shown in our 2019 study of opioid use after ACLR [4]: pain control was achieved with high patient satisfaction after outpatient ARCR with surgeon-administered analgesic block. Additionally, we found limited post-operative opioid use and no long-term opioid use in this opioid-naïve population. The protocol can be effective with a low complication rate.

One limitation of our study is reliance on patient reporting of opioid use. However, we did use our state’s prescription drug monitoring program (E-FORCSE) to ascertain whether patients were receiving opioids from outside providers. Another limitation is that we did not track patients’ ibuprofen use. The main objective was to document home opioid use. Because ibuprofen is so readily available in most homes, we felt that it could not be reliably accounted for, either in patient-reported use or in prescription monitoring. We also did not track the medications administered by the anesthesiologist in the immediate post-operative period. Controlled studies may be able to eliminate any possible discrepancies in regard to this.

In addition, we did not specifically quantify functional outcomes. Patients’ strength, range of motion, and satisfaction were assessed and met or exceeded the expectations of both patients and the senior author (G.L.C.). Large population-controlled studies may also help to further analyze the relationship between age and post-surgical opioid use. Orthopedists have been called upon to limit outpatient prescriptions of opioids to minimize misuse and diversion. This therefore was our focus, while documenting the 4-month post-operative period. It has been shown that pre-operative opioid use is a risk factor for increased post-operative use [28]. However, we purposely restricted our study to opioid-naïve patients to gain more data on this patient population, which may help in the formulation of standardized guidelines. Also, ours was not a randomized, double-blind, controlled study. Large-sample controlled studies may be useful in demonstrating the effectiveness of this block after ARCR.

The high degree and duration of post-operative pain associated with ARCR make it an ideal testing ground for alternative anesthetic protocols in shoulder surgery. One major challenge is adequately controlling pain while avoiding the risks of excessive opioid use, such as misuse, abuse, and diversion [10, 21]. ISB is widely used and is generally performed pre-operatively by the anesthesiologist using guidance and with the patient awake [1, 26]. This can increase the patient’s discomfort and anxiety before the procedure. A systematic review of single-shot ISB concluded that effective analgesia is limited to 6 to 8 h and is then associated with subsequent increases in pain and opioid use between 16 and 24 h after surgery [1]. Abdallah et al. raised the question of whether the benefit of early pain relief may be overwhelmed by the rebound pain when the block wears off.

A different strategy would be to focus on the distal nerves that innervate the shoulder. The suprascapular nerve is estimated to innervate 70% of the joint, capsule, subacromial space, and acromioclavicular joint [7]. A systematic review comparing SSNB with ISB found that no clinically meaningful differences in pain control existed at the 24-h time point [9]. Importantly, SSNB reduced the odds of ISB-related respiratory complications by 70%. In a study of the first 5 days after ARCR, patients with local injection into the suprascapular notch and perimeter of the shoulder (termed a “field block”) in addition to ISB had a 64% reduction in opioid consumption, as compared with ISB alone [20]. Additionally, 28% of patients in the group receiving ISB alone required an opioid refill, as compared with no patients in the group receiving the additional anesthesia with the field block. The sensory branches of the suprascapular nerve originate adjacent to the suprascapular notch and then travel along the base of the coracoid process to distribute to the shoulder structures [11, 14]. It was proposed that an effective and safe sensory-only block can be performed if the needle is directed to that point using external landmarks [11, 14]. The addition of an ANB has been shown to significantly lower pain scores in patients who received an SSNB [17]. We postulate that the lack of significant rebound pain crisis in our patients helped optimize pain management, thereby reducing the opioid requirement, in terms of both quantity and duration.

An ideal regional anesthetic for outpatient ARCR would be a blockade that is technically simple, has a rapid onset of action, is highly effective, has no or few adverse effects, and is relatively inexpensive. In general, anesthesiologist charges at our institutions for ISB, SSNB, or ANB are approximately $1500. In contrast, the cost of our block technique was $12 and took 2 min to perform. What differentiates this technique from others is that the surgeon uses anatomic landmarks, without ultrasound or electrodiagnostic guidance, and performs it at the conclusion of surgery, while the patient is still under general anesthesia. This strategically minimizes patient inconvenience, costs, and procedure time. Importantly, it also eliminated the need for additional PCA or anesthetic blocks. Home care after discharge was simple, with no infusion devices to manage. There were no readmissions or occurrences of significant rebound pain crisis after discharge, nor were there any late night calls to the physician or emergency center evaluations for pain. In addition, we found no suprascapular or axillary nerve deficits on physical exam during follow-up.

Pre-operative education may help to reduce opioid consumption. In one study, patients given a pre-operative video and handout detailing the risks of opioid overuse had a reduction from an average of 1308 to 768 OMEs for the first 3 months after ARCR [23]. Our pre-operative discussion with patients emphasized minimal opioid use after surgery. The growing public awareness of the opioid crisis may make educational efforts more effective. Although we were unable to quantify such an effect, we consider it likely that the office visit with our surgical team on post-operative day 1 comforted the patient, set expectations, and fostered resilience after a painful procedure.

A survey study of a large group of experts in shoulder surgery found that they initially prescribed an average of 462.5 OMEs (the equivalent of 92.4 tablets of hydrocodone 5 mg) to patients after ARCR [27]. In addition, 86.5% of those surgeons reported prescribing refills of those opioids. This is despite a regional block being used in 88% of cases [27]. After an extensive review of published data, Lovecchio et al. supported the average use of 42 tablets of hydrocodone 5 mg (210 OMEs) for the first 7 days after ARCR [13]. Although no published minimal clinically important difference in opioid use has been identified, our study’s average of 4.2 tablets (21 OMEs) represents a tenfold reduction in opioid dosage. This is among the lowest reported post-ARCR opioid prescription and use in the literature. Remarkably, more than 39% of our study patients took no opioid medication at all post-operatively. In total, the vast majority (91%) of patients were able to complete post-operative care and rehabilitation while using ten or fewer tablets of a low-dose opioid (hydrocodone/acetaminophen 5/325 mg). There were no requests for opioid prescription renewals in this prospective study group. The low opioid usage and duration were accomplished without undertreating pain, as evidenced by high patient satisfaction scores and low VAS scores. We used ibuprofen as an alternative to oral opioids because it is considered safe and low in cost [19]. Furthermore, according to a study by Oh et al., ibuprofen does not significantly inhibit tendon-to-bone healing after ARCR [16].

A study by Shah et al. demonstrated that chronic opioid use can occur after just 3 days of medication [22]. Leroux et al. reported that 9.8% of opioid-naïve ARCR patients were still filling opioid prescriptions beyond 180 days after surgery [12]. In our study, the average duration of opioid consumption was just 2.4 days. In addition, 90% of patients had discontinued opioids by post-operative day 7, and no patients were taking opioids longer than 4 weeks post-operatively. Opioid consumption was inversely proportional to age (Table 2). However, we believe this difference may have limited clinical significance with such relatively low average usage. Interestingly, all other factors such as tear size, location of surgery, procedure time, BMI, and sex did not affect opioid requirements. These data may allow standardized prescription protocols without the need to vary opioid doses on the basis of those factors. Our patient opioid demand was lower than that seen in the literature for such a potentially painful procedure. In fact, we found that a substantial proportion of patients (39%) could recover while forgoing opioids altogether. To our knowledge, this study is the first to fully quantitate complete opioid usage after outpatient arthroscopic ARCR after a surgeon-administered intra-operative SSNB and ANB local–regional block performed without imaging guidance.

In conclusion, opioid use was unexpectedly low in patients undergoing ARCR after a surgeon-administered nerve block and infiltration. With this protocol, a large proportion of patients can recover from ARCR without opioids. Our results could be useful in guiding post-operative opioid prescribing after ARCR and in lowering anesthesia costs associated with ARCR.

## Electronic supplementary material

ESM 1 (PDF 516 kb)

ESM 2 (PDF 1224 kb)

## References

[1] Abdallah FW, Halpern SH, Aoyama K, Brull R (2015). Will the real benefits of single-shot interscalene block please stand up? A systematic review and meta-analysis. Anesth Analg..

[2] Brat GA, Agniel D, Beam A (2018). Postsurgical prescriptions for opioid naive patients and association with overdose and misuse: retrospective cohort study. BMJ..

[3] Brummett CM, England C, Evans-Shields J, et al. Health care burden associated with outpatient opioid use following inpatient or outpatient surgery. *J Manag Care Spec Pharm.* 2019;25(9):973–983. 10.18553/jmcp.2019.19055 PMC10397638 31313621

[4] Caldwell GL, Selepec MA (2019). Reduced opioid use after surgeon-administered genSicular nerve block for anterior cruciate ligament reconstruction in adults and adolescents. HSS Journal..

[5] Checcucci G, Allegra A, Bigazzi P, Gianesello L, Ceruso M, Gritti G (2008). A new technique for regional anesthesia for arthroscopic shoulder surgery based on a suprascapular nerve block and an axillary nerve block: an evaluation of the first results. Arthroscopy..

[6] Dowell D, Haegerich TM (2016). Using the CDC guideline and tools for opioid prescribing in patients with chronic pain. Am Fam Physician..

[7] Fredrickson MJ, Krishnan S, Chen CY (2010). Postoperative analgesia for shoulder surgery: a critical appraisal and review of current techniques. Anaesthesia..

[8] Hughes MS, Matava MJ, Wright RW, Brophy RH, Smith MV (2013). Interscalene brachial plexus block for arthroscopic shoulder surgery: a systematic review. J Bone Joint Surg Am..

[9] Hussain N, Goldar G, Ragina N, Banfield L, Laffey JG, Abdallah FW (2017). Suprascapular and interscalene nerve block for shoulder surgery: a systematic review and meta-analysis. Anesthesiology..

[10] Labrum JT, Ilyas AM (2017). The opioid epidemic: postoperative pain management strategies in orthopaedics. JBJS Rev..

[11] Laumonerie P, Blasco L, Tibbo ME (2019). Ultrasound-guided versus landmark-based approach to the distal suprascapular nerve block: a comparative cadaveric study. Arthroscopy..

[12] Leroux TS, Saltzman BM, Sumner SA (2019). Elective shoulder surgery in the opioid naïve: rates of and risk factors for long-term postoperative opioid use. Am J Sports Med..

[13] Lovecchio F, Derman P, Stepan J (2017). Support for safer opioid prescribing practices: a catalog of published use after orthopaedic surgery. J Bone Joint Surg Am..

[14] Matsumoto D, Suenaga N, Oizumi N, Hisada Y, Minami A (2009). A new nerve block procedure for the suprascapular nerve based on a cadaveric study. J Shoulder Elbow Surg..

[15] Navarro RA, Lin CC, Foroohar A, Crain SR, Hall MP (2018). Unplanned emergency department or urgent care visits after outpatient rotator cuff repair: potential for avoidance. J Shoulder Elbow Surg..

[16] Oh JH, Seo HJ, Lee YH, Choi HY, Joung HY, Kim SH (2017). Do selective COX-2 inhibitors affect pain control and healing after arthroscopic rotator cuff repair? A preliminary study. Am J Sports Med..

[17] Park JY, Bang JY, Oh KS (2016). Blind suprascapular and axillary nerve block for post-operative pain in arthroscopic rotator cuff surgery. Knee Surg Sports Traumatol Arthrosc..

[18] Price DJ (2007). The shoulder block: a new alternative to interscalene brachial plexus blockade for the control of postoperative shoulder pain. Anaesth Intensive Care..

[19] Secrist ES, Freedman KB, Ciccotti MG, Mazur DW, Hammoud S (2016). Pain management after outpatient anterior cruciate ligament reconstruction: a systematic review of randomized controlled trials. Am J Sports Med..

[20] Sethi PM, Brameier DT, Mandava NK, Miller SR (2019). Liposomal bupivacaine reduces opiate consumption after rotator cuff repair in a randomized controlled trial. J Shoulder Elbow Surg..

[21] Seymour RB, Ring D, Higgins T, Hsu JR (2017). Leading the way to solutions to the opioid epidemic: AOA Critical Issues. J Bone Joint Surg Am..

[22] Shah A, Hayes CJ, Martin BC (2017). Characteristics of initial prescription episodes and likelihood of long-term opioid use—United States, 2006–2015. MMWR Morb Mortal Wkly Rep..

[23] Syed UAM, Aleem AW, Wowkanech C (2018). Neer Award 2018: the effect of preoperative education on opioid consumption in patients undergoing arthroscopic rotator cuff repair: a prospective, randomized clinical trial. J Shoulder Elbow Surg..

[24] Uquillas CA, Capogna BM, Rossy WH, Mahure SA, Rokito AS (2016). Postoperative pain control after arthroscopic rotator cuff repair. J Shoulder Elbow Surg..

[25] US Food and Drug Administration. *Drug Disposal: Flush Potentially Dangerous Medicine*. 2018: https://www.fda.gov/drugs/disposal-unused-medicines-what-you-should-know/drug-disposal-flush-potentially-dangerous-medicine. Updated December 19, 2018.

[26] Warrender WJ, Syed UAM, Hammoud S (2017). Pain management after outpatient shoulder arthroscopy: a systematic review of randomized controlled trials. Am J Sports Med..

[27] Welton KL, Kraeutler MJ, McCarty EC, Vidal AF, Bravman JT (2017). Current pain prescribing habits for common shoulder operations: a survey of the American Shoulder and Elbow Surgeons membership. J Shoulder Elbow Surg..

[28] Westermann RW, Anthony CA, Bedard N (2017). Opioid consumption after rotator cuff repair. Arthroscopy..

